# Role of Regulatory T Cells and Transglutaminase 2 Inhibitors in Celiac Disease: A Systematic Review

**DOI:** 10.7759/cureus.84901

**Published:** 2025-05-27

**Authors:** Ayesha Javed, Amina Saeed, Ayesha Akhtar, Amna Khalid, Amna Saleem Khan, Waseem Rabba

**Affiliations:** 1 Pediatric Medicine, M. Islam Teaching Hospital, Gujranwala, PAK; 2 Obstetrics and Gynecology, Bhatti International Teaching Hospital, Kasur, PAK; 3 General Internal Medicine, Avicenna Medical College, Lahore, PAK; 4 Medicine, Christus Santa Rosa Hospital, New Braunfels, USA; 5 General Medicine, Pakistan Air Force (PAF) Hospital Islamabad, Islamabad, PAK; 6 Health Sciences, McMaster University, Hamilton, CAN

**Keywords:** celiac disease, drug therapy, regulatory t cells, tg2, transglutaminase 2 inhibitor

## Abstract

Celiac disease is an autoimmune disorder triggered by the ingestion of gluten in genetically predisposed individuals, leading to chronic intestinal inflammation and damage to the small intestinal lining (villus atrophy). While a strict gluten-free diet remains the primary treatment, emerging therapies targeting the immune response offer promising alternatives. This review focuses on the therapeutic potential of transglutaminase 2 (TG2) inhibitors, enzymes that, when overactive, contribute to immune system attacks on the gut, and regulatory T cells (Tregs), specialized immune cells that help calm down excessive immune reactions. This systematic review followed the Preferred Reporting Items for Systematic reviews and Meta-Analyses guidelines. Literature was searched across PubMed, Embase, and the Cochrane Library using both text terms and controlled vocabulary with Boolean operators (“AND,” “OR”). We included full-text, open-access, English-language articles published between 2005 and 2025. The methodological quality of studies was assessed using the Mixed Methods Appraisal Tool. A total of 68 articles were initially identified. After screening and applying inclusion criteria, 14 studies were included in the final analysis. Among them, eight studies were rated as high quality (low risk of bias), while six were of moderate quality (uncertain risk of bias). TG2 inhibitors showed promising effects such as improved intestinal structure (villous architecture), reduced gastrointestinal symptoms, stabilized immune cell levels in the gut, and decreased activation of gluten-specific immune cells (CD4+ T cells). Treg therapies also demonstrated the ability to reduce inflammation by limiting the production of harmful immune signals such as interferon-gamma and interleukin-21. The findings highlight the therapeutic potential of TG2 inhibitors and Treg-based treatments in managing celiac disease by directly targeting the immune response. While preliminary results are promising, further clinical research is needed to confirm their effectiveness and safety for routine clinical use.

## Introduction and background

Celiac disease presents as an autoimmune intestinal disease as gluten interacts with genetically susceptible people [1]. Gluten is a dietary staple that is a leading environmental trigger for the development of celiac disease. Patients with celiac disease show hypersensitivity toward cereal gluten proteins, leading to lesions within the digestive system [2]. This complex disorder develops due to genetic vulnerability and environmental contributors [3]. Research data regarding the prevalence of the disease now exists for every part of Earth, excluding Antarctica [4]. According to research, 0.5% to 2% of people in the general population have celiac disease, yet the worldwide average indicates 1% are affected [5].

Regulatory T cells (Tregs) are a type of immune cell that help control inflammation and prevent the immune system from attacking the body’s own tissues. Transglutaminase 2 (TG2) is an enzyme involved in tissue repair, but in certain diseases, such as celiac disease, it can trigger harmful immune reactions. Simply put, Tregs act as the immune system’s “brakes,” while TG2 can accidentally push the “gas pedal” in autoimmune conditions [6]. Studies have shown celiac disease in 10% of high-risk populations comprising children with family members affected by celiac disease, as well as patients with type 1 diabetes and IgA deficiency, alongside individuals with chromosomal disorders [7]. A diagnosis of celiac disease needs confirmation from positive serology involving IgA anti-TG2 and anti-endomysial antibodies, together with villous atrophy observed via a small intestinal biopsy [8]. The evaluation of Tregs and TG2 inhibitors as therapeutic options is grounded in their critical roles in immune regulation and disease pathology. Tregs are key mediators of immune tolerance, capable of suppressing aberrant immune responses that drive chronic inflammation and autoimmunity. Therefore, enhancing Treg function or numbers can restore immune balance and reduce tissue damage [9]. TG2, on the other hand, is an enzyme involved in the post-translational modification of proteins and has been implicated in promoting inflammation, fibrosis, and autoantigen formation in various diseases. Inhibiting TG2 activity may interrupt these pathological processes, thereby limiting disease progression [10].

Together, targeting Tregs and TG2 represents a dual strategy, i.e., modulating the immune system to prevent excessive inflammation and directly blocking enzymatic pathways that contribute to tissue injury. This combined approach holds promise for conditions where current treatments are insufficient, making the investigation of Tregs and TG2 inhibitors both relevant and timely [11]. Tregs function as a vital mechanism to fight inflammation, which occurs in patients with celiac disease [12]. The intestinal mucosa of affected individuals contains two main regulatory T-cell subsets, including CD4⁺ type 1 regulatory T cells (Tr1) together with CD4⁺CD25⁺Foxp3⁺ T cells (Foxp3⁺ Tregs) [12]. The anti-inflammatory effect of these cells functions through cytokine release mechanisms that suppress harmful immune responses to gliadin compound gluten by producing both interleukin (IL)-10 and transforming growth factor-beta cytokines [13].

The critical function of TG2 in the pathogenesis of celiac disease involves gluten peptide deamidation, through which it promotes antigenic presentation along with increased gluten-reactive T cells [14]. Research shows that blocking TG2 represents an active approach for treating celiac disease [15]. Pharmaceutical researchers have evaluated ZED1227 as a possible therapeutic agent for celiac disease due to its blocking function against TG2-mediated deamidation and crosslinking of gliadin peptides [16]. When binding the active state of TG2, the compound establishes a stable covalent bond with the cysteine present in the catalytic center [17].

Primary prevention of celiac disease depends on infant feeding methods for gluten introduction, including breastfeeding choice at exposure time and the quantity of gluten, as well as the role of intestinal infections and delivery methods, antibiotic use, and gut microbiota makeup [18]. Screening high-risk populations and population-wide testing allow healthcare professionals to identify individuals who currently have or may develop the disease [19]. Tertiary prevention includes two main components, namely, proper gluten-free diet management and monitoring gluten immunogenic peptides through dietary interviews and serology and duodenal biopsies, as well as considering further treatments such as larazotide acetate endopeptidases and desensitization therapy [20,21].

The consumption of gluten activates chronic autoimmune reactions in the small intestine, which cause immune damage to patients with celiac disease. The immune-tolerating function of Tregs is essential for celiac disease, yet their role in disease pathogenesis remains undefined. The immune mechanisms of the TG2 enzyme, which induces inflammation during the development of celiac disease, remain unclear regarding its sensitivity to potential inhibitors. The interaction between Tregs and TG2 inhibition during immune responses in celiac disease requires further investigation despite individual research on Tregs and TG2 dysregulation in the pathogenesis of celiac disease. Drugs targeting TG2 and Tregs have shown promising findings in animal studies, yet no sufficient clinical evidence exists regarding their long-term combined benefits or effectiveness. This review combines existing research on the role of Tregs and TG2 in celiac disease to assess therapeutic outcomes from blocking TG2 activity, evaluate the use of Tregs for treatment, and develop new management methods for celiac disease.

## Review

Methodology

This review followed the requirements established by the Preferred Reporting Items for Systematic reviews and Meta-Analyses (PRISMA) guidelines [22]. The research question was formulated using the PICO framework [23]. Table 1 describes the PICO framework specifying the vital research elements comprising Population, Intervention, Comparison, and Outcome. The PICO framework makes data extraction more efficient and produces better systematic reviews with relevant studies.

**Table 1:** PICO framework.

Concepts	Text words	Controlled vocabulary
Population/Problem: patients with celiac disease	“Celiac Disease,” “Gluten Intolerance,” “Autoimmune Enteropathy”	“Celiac Disease” [MeSH]
Intervention: transglutaminase 2 inhibitor	“TG2 inhibitors,” “Transglutaminase 2 inhibitors”	“Transglutaminase” [MeSH]
Comparative: regulatory T cells	“Treg Therapy,” “Regulatory T Cells”	“Regulatory” [MeSH] “T-Lymphocytes” [MeSH]
Outcomes	“Immune regulation,” “disease remission,” “intestinal damage,” “immune tolerance”	“Immune Tolerance” [MeSH], “Intestinal Mucosa” [MeSH], “Inflammation” [MeSH]

Research Question

What is the role of Tregs and TG2 inhibitors for celiac disease?

Search Strategy and Search Terms

We conducted systematic searches on PubMed, Embase, and Cochrane databases using a combination of keywords and controlled vocabulary terms, including “Transglutaminase 2 Inhibitor,” “Regulatory T Cells,” “TG2,” “Tregs,” “Immune Regulation,” “Intestinal Damage,” and “Immune Tolerance.” Boolean operators “AND” and “OR” were applied in various combinations. The search string was (“Transglutaminase 2 Inhibitor” OR “Regulatory T Cells”) AND (“Celiac Disease” OR “Coeliac Disease” OR “Gluten-sensitive enteropathy”) AND (“Intestinal Damage” OR “Immune Tolerance” OR “Adverse reaction” OR “quality of life”). Limiters were applied to include only open-access, full-text, English-language articles published between 2014 and 2025 involving human subjects.

Inclusion and Exclusion Criteria

We included only experimental studies to focus on evidence that could demonstrate causality or therapeutic effect and excluded observational designs that are less able to determine treatment efficacy. Adult patients of both genders were included to generalize findings across the typical adult population affected by celiac disease. The outcomes of interest, such as the villus height to crypt depth ratio (VH:CrD) and intraepithelial lymphocyte density (IEL), are standard histological markers of intestinal damage and repair in celiac disease, while health-related quality of life (HRQoL) is critical for assessing clinical benefit beyond biological measures. We limited the inclusion to the last two decades for comprehensive coverage, but prioritized the most current research by focusing on recent years. Studies had to be accessible (open access) and fully available in the English language to allow thorough data extraction and appraisal.

We excluded studies that could introduce heterogeneity or skew the review’s focus, such as observational studies, case reports, and reviews, which may provide weaker evidence or secondary analyses. Non-adult populations were excluded because immune regulation and disease progression may differ significantly in children and adolescents. Studies published before 2005 were excluded due to limitations in data availability and possible differences in diagnostic or therapeutic standards, ensuring the review reflects contemporary practice.

Study Selection Process

Two independent reviewers first screened titles and abstracts, followed by the full text, to reduce selection bias. Independent review minimizes subjective influence and improves reliability. Disagreements were resolved by consensus, ensuring that all decisions were carefully considered and justified. Only studies meeting all the inclusion criteria and available in full text were included, ensuring comprehensive evaluation and data extraction [24].

Methodological Quality Assessment

We employed the Mixed Methods Appraisal Tool (MMAT), a validated instrument suitable for assessing a variety of study designs. MMAT scores range from 1 to 5, where a score of 5 indicates low risk of bias (high methodological quality), 4 indicates medium risk, and lower scores suggest higher risk or unclear quality. This quality assessment allows readers to gauge the reliability of the included studies and ensures that the synthesis accounts for potential limitations. It also helps identify areas where evidence may be weaker or inconsistent [25].

Data Extraction and Synthesis

A data sheet was created to collect extracted data from the included studies for the synthesis of study findings. We collected basic information, such as study design, demographic characteristics, and characteristics related to the outcome of interest, including objectives, intervention protocol, outcome, study findings, and comparative effectiveness. Subsequently, a thematic analysis using an inductive, data-driven approach was employed to analyze the data sheet [26]. Then, an iterative approach was applied for a further in-depth investigation and convergence of the results [27]. Finally, the studies were analyzed critically to synthesize the evidence, ensuring the practice was evidence-based.

Results

**Figure 1:**
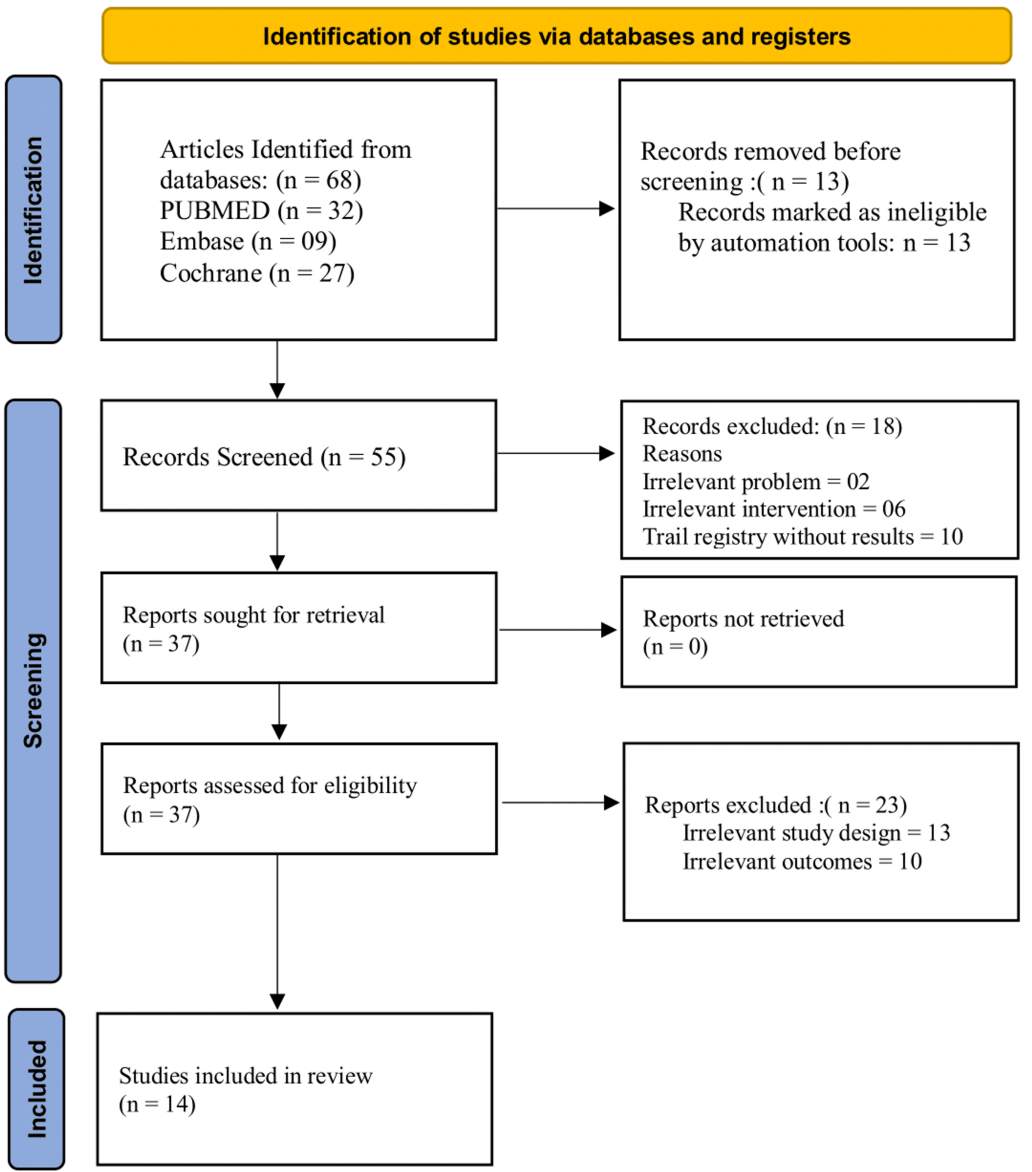
Preferred Reporting Items for Systematic reviews and Meta-Analyses (PRISMA) flowchart.

The PRISMA guidelines were followed to systematically synthesize the evidence presented in this review. In total, 68 articles were retrieved during the initial search using keywords, text words, and controlled vocabulary on PubMed, Embase, and the Cochrane Library databases. A total of 13 duplicate articles were removed using EndNote. Then, 55 articles were selected for screening. Of these, 18 irrelevant articles were removed based on title and abstract reading. The eligibility of 37 articles was determined through pre-specified criteria. Further, 23 irrelevant articles were excluded through a thorough review of the studies. After the eligibility check, only 14 articles were selected for quality assessment. Figure 1 presents the PRISMA flowchart.

Table 2 presents the risk of bias using the MMAT. An MMAT score of 4 indicates a medium risk of bias, and an MMAT score of 5 indicates a low risk of bias. Low risk implies that no significant issues related to bias were noted in the study. On the other hand, high risk implies potential biases that may impact the internal validity of the studies. Finally, an unclear score implies a lack of sufficient information to assess the risk of bias.

**Table 2:** Mixed Methods Appraisal Tool scores.

Study	MMAT score	Selection bias	Performance bias	Detection bias	Attrition bias	Reporting bias	Other bias	Overall risk of bias
Dotsenko et al., 2024 [14]	4	Low	Low	High	Low	Unclear	None	Moderate
Schuppan et al., 2021 [15]	4	Low	Low	High	Low	Unclear	None	Moderate
Ray et al., 2021 [28]	4	Low	Low	High	Low	Unclear	None	Moderate
Isola et al., 2023 [29]	5	Low	Low	Low	Low	Low	None	Low
Dotsenko et al., 2022 [30]	5	Low	Low	Low	Low	Low	None	Low
Rauhavirta et al., 2013 [31]	5	Low	Low	Low	Low	Low	None	Low
Gianfrani et al., (2023) [32]	5	Low	Low	Low	Low	Low	None	Low
Porret et al., 2025 [33]	4	Low	Low	High	Low	Unclear	None	Moderate
Maiuri et al., 2005 [34]	4	Low	Low	High	Low	Unclear	None	Moderate
Cook et al., 2017 [35]	5	Low	Low	Low	Low	Low	None	Low
Christophersen et al., 2021 [36]	5	Low	Low	Low	Low	Low	None	Low
Han et al., 2013 [37]	4	Low	Low	High	Low	Unclear	None	Moderate
Risnes et al., 2024 [38]	5	Low	Low	Low	Low	Low	None	Low
Granzotto et al., 2009 [39]	5	Low	Low	Low	Low	Low	None	Low

The included studies demonstrated varying levels of methodological quality based on the MMAT scores and risk of bias domains. A total of seven studies, including those by Schuppan et al. (2021), Dotsenko et al. (2024), Ray et al. (2021), Porret et al. (2025), Maiuri et al. (2005), Han et al. (2013), and others, had an MMAT score of 4 and were rated as having a moderate overall risk of bias, primarily due to high detection bias and unclear reporting bias. In contrast, the remaining studies, including those by Isola et al. (2023), Dotsenko et al. (2022), Rauhavirta et al. (2013), and Christophersen et al. (2021), scored 5 on the MMAT and demonstrated low risk of bias across all domains, indicating strong methodological rigor. Most notably, performance and selection biases were consistently rated as low across all studies, while detection bias was the most frequent source of methodological concern [14,15,28-39].

Table 3 presents the characteristics and findings of studies included in the review. Studies indicated that TG2 inhibitors have led to reported improvement in villous architecture, reduction in gastrointestinal symptoms, and stabilization of IEL counts. It significantly reduced gluten-specific CD4+ T-cell activation in the lamina propria, which is central to the pathogenesis of celiac disease. This suppression of pathogenic T-cell responses contributes to decreased production of pro-inflammatory cytokines, such as interferon-gamma (IFN-γ) and IL-21, resulting in reduced mucosal inflammation. Research demonstrates therapeutic possibilities of Treg therapy and TG2 inhibitor use to treat celiac disease. New treatment possibilities emerge when immune response regulation occurs through these methods, according to available evidence. Further clinical research is needed to validate their clinical effectiveness and safety standards for the management of celiac disease.

**Table 3:** Characteristics and findings of the studies included in the review.

Author/Year	Objectives	Interventional protocol	Outcome measured	Findings	Comparative effectiveness
Dotsenko et al., 2024 [14]	To evaluate the efficacy of the TG2 inhibitor ZED1227 by performing transcriptional analysis of duodenal biopsies from individuals with celiac disease	100 mg per day ZED1227 or placebo for 6 weeks, combined with a gluten challenge in participants on a long-term gluten-free diet	Molecular-level changes in gene expression	ZED1227 treatment effectively prevented gluten-induced intestinal damage and inflammation. Nearly half of the gluten-induced gene expression changes were associated with the epithelial interferon-γ response	ZED1227 was more effective than a placebo in preserving mucosal integrity and reducing inflammation in response to gluten
			Transcriptome analysis of duodenal biopsies	Preserved transcriptome signatures related to mucosal morphology, inflammation, cell differentiation, and nutrient absorption, comparable to the gluten-free diet group	
Schuppan et al., 2021 [15]	To assess the efficacy and safety of ZED1227 in attenuating gluten-induced mucosal damage	Six-week treatment with ZED1227 at three dose levels (10 mg, 50 mg, and 100 mg) or placebo, with a daily gluten challenge	Attenuation of gluten-induced duodenal mucosal damage (villus height to crypt depth ratio)	Villus height to crypt depth ratio	ZED1227 at all dose levels was more effective than placebo in reducing gluten-induced mucosal injury
				10 mg group: 0.44 (95% CI = 0.15 to 0.73; p = 0.001)	
				50 mg group: 0.49 (95% CI = 0.20 to 0.77; p < 0.001)	
				100 mg group: 0.48 (95% CI = 0.20 to 0.77; p < 0.001)	
			Intraepithelial lymphocyte density	Intraepithelial lymphocyte density reduction	
				10 mg group: -2.7 cells per 100 epithelial cells (95% CI = -7.6 to 2.2)	
				50 mg group: -4.2 cells per 100 epithelial cells (95% CI = -8.9 to 0.6)	
				100 mg group: -9.6 cells per 100 epithelial cells (95% CI = -14.4 to -4.8)	
			Health-related quality of life (QoL)	The 100-mg dose also improved symptoms and quality of life	
Ray et al., 2021 [28]	To evaluate the efficacy and safety of the selective oral TG2 inhibitor ZED1227 in reducing gluten-induced duodenal mucosal damage in patients with celiac disease	Daily gluten challenge + ZED1227 (three dose groups: 10 mg, 50 mg, 100 mg). Duration: 6 weeks. Comparator: placebo	Duodenal mucosal injury	All doses of ZED1227 significantly reduced gluten-induced mucosal injury compared to placebo	All three doses were effective compared to the placebo
Isola et al., 2023 [29]	To study the accumulation and localization of orally administered ZED1227 in the small intestinal mucosa of celiac disease patients and its effect on TG2	Oral administration of ZED1227 during gluten challenge in celiac disease patients; comparison to the placebo group	Localization of ZED1227-TG2 complex in intestinal tissue	ZED1227-TG2 signal was:	Compared to the placebo, ZED1227 was shown to inhibit TG2 activity and accumulate at specific sites (villus epithelium), whereas the placebo showed no specific staining
				Strongest at luminal epithelial brush border	
				Absent in the placebo	
				Only 20% as strong in lamina propria	
Dotsenko et al., 2022 [30]	To evaluate the efficacy and safety of ZED1227, a selective TG2 inhibitor, in preventing gluten-induced intestinal mucosal damage and improving symptoms in celiac disease patients undergoing a controlled gluten challenge	Six-week treatment with oral ZED1227 (10 mg, 50 mg, or 100 mg daily) vs. placebo, during a daily 3 g gluten challenge. All patients had celiac disease, were HLA-compatible, and had maintained a gluten-free diet for ≥12 months	Villous height to crypt depth ratio (VH:CrD)	VH:CrD improved significantly in all ZED1227 groups vs. placebo	All ZED1227 doses showed superior efficacy over placebo in preventing gluten-induced mucosal injury and improving symptoms and QoL, with 100 mg being the most effective
			Symptom severity (CSI)	Estimated symptom score differences (CSI):	
				10 mg: –3.0 (p = 0.0384)	
				50 mg: –2.0 (p = 0.1715)	
				100 mg: –3.8 (p = 0.0095)	
			quality of life (CDQ),	CDQ QoL score improvements:	
				10 mg: 5.3 (p = 0.0659)	
				50 mg: 2.9 (p = 0.3062)	
				100 mg: 5.8 (p = 0.0474)	
Rauhavirta et al., 2013 [31]	To evaluate whether TG2 inhibitors (R281 and R283) can prevent the toxic effects of gliadin in vitro (Caco-2 cells) and ex vivo (celiac patient biopsy cultures)	PT-gliadin (peptic-tryptic-digested gliadin) exposure. Co-treatment with cell-impermeable R281 or cell-permeable R283 TG2 inhibitors	Transepithelial resistance, cytoskeleton changes, junction protein expression, ERK1/2 phosphorylation	Both inhibitors reduced gliadin-induced damage in Caco-2 cells	R281 (cell-impermeable) showed stronger inhibition of gliadin-induced toxicity compared to R283 (cell-permeable)
				R281 showed slightly more potent effects	
				R281 reduced inflammatory and proliferative markers in biopsies but did not reduce TG2 autoantibody secretion	
			CD25+, IL-15+, FOXP3+ cells, Ki-67+ cells, TG2-autoantibody secretion	TG2 inhibitors, especially R281, can mitigate gliadin-induced immune and epithelial responses, supporting their potential as therapeutic agents in celiac disease	
Gianfrani et al., 2023 [32]	To investigate whether gliadin-specific type 1 regulatory T (Tr1) cells can be isolated from the intestinal mucosa of celiac disease patients in remission	Stimulation of celiac disease biopsies with gliadin in the presence or absence of IL-10	Proliferation and cytokine production of gliadin-specific T cells	Stimulation with gliadin and IL-10 suppressed antigen-specific proliferation and cytokine production of pathogenic T cells	Gliadin-specific Tr1 cells showed the ability to suppress pathogenic Th0 cells, suggesting their potential for therapeutic intervention to restore immune homeostasis in celiac disease
			Detection of gliadin-specific Tr1 cell clones and their suppressive effects on pathogenic T cells	Gliadin-specific Tr1 cells were anergic, restricted by DQ2 (CD-associated HLA), and produced IL-10 and IFN-γ, but little or no IL-2 or IL-4, suppressing the proliferation of pathogenic Th0 cells	
Porret et al., 2025 [33]	To evaluate the number and suppressive function of regulatory T cells (CD4+CD25+FOXP3+) in celiac disease patients compared to healthy controls	Comparison of percentage and marker expression of CD4+CD25+FOXP3+ Tregs in peripheral blood of celiac disease patients and healthy donors	Frequency and FOXP3 expression of Tregs.	Significant impairment in the suppressive function of Tregs in celiac disease patients (p = 0.00)	Tregs from healthy individuals suppressed T-cell proliferation more effectively than those from celiac disease patients
Maiuri et al., 2005 [34]	To assess whether inhibiting TG2 can mitigate gliadin-induced toxicity in celiac disease through ex vivo and cell culture models	Duodenal biopsies and T84 cells were exposed to gliadin peptides with/without TG2 inhibitors. Cultures were examined at 20 minutes, 3 hours, and 24 hours	TG2 activity	TG2 inhibition controlled T-cell activation but not all mucosal responses	Inhibiting the enzymatic activity of TG2 limits immune activation. Targeting surface TG2 binding offers a broader protective effect, potentially superior
			Actin cytoskeleton changes	Binding of surface TG2 inhibited T-cell activation and p31-43-induced epithelial alterations (actin rearrangement, phosphorylation, and apoptosis)	
Cook et al., 2017 [35]	To determine whether patients with celiac disease exhibit dysfunction or a lack of gluten-specific FOXP3+ Treg cells	Oral wheat challenge to stimulate recirculation of gluten-specific T cells; peripheral blood collected before and after challenge	Frequency and function of gluten-specific FOXP3+CD39+ Treg cells	Approximately 80% of circulating gluten-specific CD4+ T cells were FOXP3+CD39+ Treg cells	Gluten-specific Treg cells vs. polyclonal Treg cells: - Polyclonal Tregs retained normal function, while gluten-specific Tregs showed impaired suppressive activity
				These Treg cells showed significantly reduced suppressive function	
			Suppressive function compared to polyclonal Treg cells	Normal suppressive function observed in polyclonal peripheral Treg cells	
Christophersen et al., 2021 [36]	To characterize how gluten-specific CD4+ T cells and celiac disease-associated CD8+ and γδ+ T cells change phenotypically in treated celiac disease patients upon gluten exposure, and to identify potential markers for T-cell-directed therapy	Three-day gluten challenge in treated celiac disease patients, followed by immunophenotyping of T cells on day 6	Phenotypic changes in gluten-specific CD4+ T cells and celiac disease-associated CD8+/γδ+ T cells	Gluten-specific CD4+ T cells acquired expression of CD147, CD70, PD-1, ICOS, CD28, CD95, CD38, and CD161 after gluten exposure	Gluten challenge revealed that phenotypic changes in CD4+ T cells mirror those seen in CD8+/γδ+ T cells associated with active disease, suggesting shared pathogenic mechanisms and informing therapeutic target development
				Day 6 specific markers: CXCR6, CD132, CD147	
				Stable markers: integrin α4β7, CCR9, CXCR3; 52% CXCR5+ at baseline; 0% CXCR5+ on day 6	
				Overlap in phenotype with CD38+ and CD103+ CD8+/γδ+ T cells on day 6	
Han et al., 2013 [37]	To investigate whether gluten exposure induces antigen-driven activation of CD8+ αβ and γδ T cells in celiac disease, alongside gluten-specific CD4+ T-cell responses	Gluten exposure in celiac patients on a gluten-free diet; subsequent analysis of peripheral blood T-cell populations	Activation and appearance of gut-homing CD8+ αβ and γδ T cells	Gluten exposure leads to the appearance of activated, gut-homing CD8+ αβ and γδ T cells	Revealed that not only CD4+ but also CD8+ αβ and γδ T cells are antigen-activated during gluten exposure, highlighting their coordinated role in celiac disease pathogenesis
			TCR repertoire diversity and specificity in response to gluten	TCR sequencing revealed highly focused, antigen-driven repertoires	
				Coordinated immune response involving CD4+, CD8+, and γδ T cells	
Risnes et al., 2024 [38]	Investigation into how gluten-specific CD4+ T cells transition into memory T cells and how this correlates with clinical remission markers during the first year of a gluten-free diet	Patients were followed for 12 months during adherence to a gluten-free diet. Assessed T-cell activation and memory transition via flow cytometry, histology, symptom scores, and serology	Frequency and phenotype of gluten-specific CD4+ T cells	Gluten-specific T cells peaked in blood at day 14 and decreased within 10 weeks. Markers CD38, ICOS, HLA-DR, Ki-67 decreased within 3 days	A gluten-free diet leads to rapid immunological and serological normalization and the development of a persistent memory T-cell phenotype, revealing a window for immunomodulatory interventions in early celiac disease
				PD-1, CD39, and OX40 persisted up to 12 months	
			Disease-specific serology (e.g., IgA–IgA-transglutaminase 2)	IgA–TG2 levels decreased significantly within 4 weeks	
Granzotto et al., 2009 [39]	To investigate whether defects in the number or function of Tregs contribute to the pathogenesis and autoimmunity seen in celiac disease	Comparison of Treg frequency and expression of regulatory markers (FOXP3). Functional suppression assays using autologous responder T cells	Frequency and FOXP3 expression of CD4+CD25+ Tregs	No significant difference in the percentage or FOXP3 expression of Tregs between celiac disease patients and controls	Demonstrates functional impairment of Tregs in celiac disease versus healthy controls despite similar cell counts and marker expression
			- Suppressor activity of Tregs on CD4+CD25− T cells	Suppressive function significantly impaired in Tregs from celiac disease patients	

Villus Height to Crypt Depth Ratio

Assessment of mucosal damage in celiac disease utilizes VH:CrD ratio as a vital histological indicator for clinicians. The ratio provides information about intestinal health, as higher levels show healthy architecture, while decreased values occur when villous atrophy develops, such as in celiac disease. In the study by Schuppan et al., ZED1227 proved effective against VH:CrD deterioration across all treatment amounts while delivering optimum performance with an odds ratio of 0.49 (95% confidence interval (CI) = 0.20-0.77) in the 50 mg dose group versus placebo [15]. Dotsenko et al. established that ZED1227 treatment led to essential improvements in VH:CrD histological results within gluten challenge trials. The experimental findings demonstrated that TG2 inhibitors successfully guard against tissue injury caused by gluten consumption [30]. According to the study by Ray et al., all TG2 inhibitor doses provided significant protection to mucosal tissues [28]. The data collected by Dotsenko et al. showed that TG2 inhibitors maintained essential molecular markers linked to healthy mucosa organization [14].

Intraepithelial Lymphocyte Density

Reduced IEL density indicates that immune activation occurs in the epithelium, as this marker is a fundamental feature of gluten-induced inflammation in celiac disease. Schuppan et al. established that treatment with ZED1227 created a dose-dependent reduction in IEL counts, reaching its peak at 100 mg, which decreased IEL counts by -9.6 cells per 100 epithelial cells (95% CI = -14.4 to -4.8)[15]. According to Dotsenko et al., the use of ZED1227 led to measurable decreases in IEL populations, reinforcing the anti-inflammatory properties of ZED1227 [30]. Dotsenko et al. acknowledged how ZED1227 protects epithelial genes along with IFN-γ-related responses. The drug ZED1227 shows dual action by decreasing both gluten-induced mucosal alteration damage and its associated inflammatory markers [15].

Health-Related Quality of Life

Celiac disease patients face severe quality of life decline because their diet restrictions coincide with persistent symptoms. TG2 inhibitors at a dose of 100 mg showed better clinical metrics than placebo in a study reported by Schuppan et al., while simultaneously boosting patients’ HRQoL [15]. Ray et al. concluded that Celiac Disease Questionnaire (CDQ) scores using celiac drug therapy at a 100 mg dose increased by 5.8 points, which achieved statistical significance with a p-value of 0.0474. TG2 inhibitors benefit patient health outcomes while providing histological and immunological benefits for patients [28].

Symptom Severity (Celiac Symptom Index)

Among critical outcome evaluation measures, the intensity of symptoms is vital for patient need assessment. The Celiac Symptom Index (CSI) from Dotsenko et al. is a quantitative tool to track dysfunction changes during gluten consumption assessments [15]. The TG2 inhibitor achieved a major symptom reduction when administered at 100 mg, resulting in a -3.8 point decrease (p = 0.0095). The 50 mg treatment group experienced a non-significant symptom decrease of -2.0 scores (p = 0.1715). According to Isola et al., greater dosage strengths produced better symptom improvement outcomes. Testing demonstrated that the drug led to healing outcomes comparable to histological progress and immunological adaptations, which confirmed its all-encompassing therapeutic ability for active celiac disease [29].

Serum Biomarkers (Anti-tTG and Anti-DGP Antibodies)

Based on Schuppan et al., the medical evaluation of celiac disease examines tissue transglutaminase antibodies (anti-tTG) and deamidated gliadin peptide antibodies (anti-DGP) as they help determine disease state and measure patient gluten consumption. The brief gluten challenge caused minimal changes in antibody numbers, although the TG2 inhibitor successfully blocked increased anti-tTG antibody concentrations. Rauhavirta et al. observed maintained stability of anti-DGP IgG titers in patients who received TG2 inhibitor treatment [31]. Gianfrani et al. reported that TG2 inhibitors act as a preventive measure by stopping gluten from triggering antibody elevation [32]. Porret et al. reported that TG2 inhibitors maintain serologic stability for gluten exposure tolerance following accidental gluten exposure [33].

Gene Expression and Transcriptomic Modulation

Dotsenko et al. reported that TG2 inhibitor administration preserved the expression of both epithelial cells and goblet cells during gluten contact [14]. Maiuri et al. concluded that the application of TG2 inhibitor diminished IFN-γ immune reactions and elevated protective gene expression levels that preserve epithelial barrier integrity. TG2 inhibitor treatment showed its most potent effects through clinical and histological responses at the maximum dosage tested. The observed changes in gene expression show that TG2 inhibitors can work through genomic pathways to develop new therapeutic options for celiac disease that extend beyond typical symptom relief and structural advantages [34].

Intraepithelial Lymphocyte Count

Research indicates that the defining marker of active celiac disease is intraepithelial lymphocytosis. Dotsenko et al. tested IEL counts in placebo patients whose numbers greatly increased. Those taking a TG2 inhibitor showed minimal variation in their IEL counts according to the findings reported by Schuppan et al. and Dotsenko et al. [14,15,30]. Christophersen et al. reported that the medication minimized gluten-triggered mucosal inflammation throughout the body. The significant decrease in IEL infiltration in the high-dose treatment group tests IEL count reliability as an effective biomarker for mucosal immune activities during assessment [36].

Intestinal Permeability (Lactulose/Mannitol Ratio)

The performance of intestinal barriers is reduced in patients with celiac disease. Researchers, including Dotsenko et al., along with other groups, utilized the lactulose/mannitol (L/M) test to evaluate intestinal permeability levels [14]. Han et al. concluded that TG2 inhibitor-treated patients demonstrated either improved or maintained their L/M permeability ratios during gluten test periods, indicating active, intact intestinal barriers. Better scores in permeability testing support mucosal health restoration and symptom reduction with immunological activation because they demonstrate TGM2 blockade and barrier functioning as the drug’s mechanism [37].

Discussion

The current research studied both Tregs and TG2 inhibitors as potential treatments for celiac disease. According to Schuppan et al., all doses of TG2 inhibitors produced better VH:CrD results than placebo [15]. Risnes et al. reported that intestinal structure protection occurred in celiac disease patients who received 50 mg of TG2 inhibitor, with an odds ratio of 0.49 (95% CI = 0.20-0.77) [38]. According to Dotsenko et al. and Ray et al., TG2 inhibitors demonstrated clinically significant improvements in VH:CrD among test subjects despite every dosage level showing tissue protection [28,30]. The study by Dotsenko et al. demonstrated that TG2 inhibitors safeguarded the genetic expressions that maintain mucosal integrity [14]. According to Granzotto et al., most celiac disease patients under medical control following a gluten-free diet displayed Marsh 3a or 3b duodenal damage [39]. According to Risnes et al., duodenal biopsies of the third part had elevated VH:CrD ratios compared with bulb samples (2.1:1.4; p < 0.0001) [38]. Daveson et al. concluded that the measured values for paired villus height and crypt depth established a strong statistical connection, which yielded an R² of 0.82 and a p-value of less than 10¹⁰. Most patients who follow a gluten-free diet still exhibit ongoing villus atrophy based on Marsh 3a and 3b injury stages according to quantitative histology investigations [40].

Schuppan et al. demonstrated that TG2 inhibitors reduced IEL density in a dose-dependent manner at the 100 mg dosage, leading to a -9.6 cells/100 epithelial cells reduction (95% CI = -14.4 to -4.8) [15]. A study conducted by Dotsenko et al. confirmed that TG2 inhibitors lead to decreased IEL density, while expanding evidence supports their capability to decrease gluten-triggered inflammation [30]. Molecular evidence from Dotsenko et al. demonstrated that treatment protected epithelial genes, while it reduced the activity of IFN-γ-mediated responses [14]. Isola et al. presented an in-depth understanding of celiac disease-related IEL function by showing that aberrant IEL behavior causes epithelial compromise, resulting in villous atrophy. IEL activation management therapy reported in the study presents a potential disease progression-alleviating technique without requiring specific therapies, yet enhances the anti-inflammatory properties of TG2 inhibitors [29]. Jabri et al. highlighted that controlling IEL activation is vital for celiac disease treatment and showed how TG2 inhibitors lower IEL counts to protect mucosal tissue structure [41].

In the study by Isola et al., the 100 mg dose of a TG2 inhibitor led to meaningful improvements in HRQoL as it increased CDQ scores by 5.8 points (p = 0.0474) over the placebo, thus demonstrating beneficial effects on patient well-being [29]. Employing structural equation modeling (SEM), Al-Sunaid et al. revealed that adherence to diet as well as symptom severity (Gastrointestinal Symptom Rating Scale (GSRS) score) and education level affected HRQoL, especially the Physical Component Summary (PCS) and Mental Component Summary (MCS) of the 36-Item Short Form Survey in a statistically significant manner. The GSRS score acted as an intermediary factor that linked dietary adherence to both PCS and MCS scores [42]. The studies agree about dietary importance, but Al-Sunaid et al. and Nikniaz et al. confirmed that physician interventions directly impact results compared to the study by Al-Sunaid et al., which focused on lifestyle adjustments and symptom control [42,43].

Rauhavirta et al. concluded that TG2 inhibitors protected epithelial and goblet cell gene expression under gluten insult by maintaining typical patterns, with the high-dose treatment group showing the most enhanced effect [31]. According to Porret et al., treatment with the drug lowered IGN-γ-mediated immune response activation while restoring barrier functions between epithelial cells at the genomic level [33]. Simpson et al. showed that the intervention stopped gluten from creating intestinal damage together with inflammation through inhibition of TG2 that underlies the pathogenesis of celiac disease [44]. Maiuri et al. supported the findings by indicating that TG2 inhibitors protected the transcriptional patterns associated with intestinal structure integrity and immune activity, as well as digestive function, while its actions specifically targeted IFN-γ signaling in epithelial cells [34]. Cook et al. produced collective evidence demonstrating that TG2 inhibitors have the potential to become a viable targeted treatment solution for celiac disease management based on molecular research [35]. Christophersen et al. reported that TG2 inhibitors were associated with regulation of gene expression, as well as significant histological and clinical improvements [36].

Clinical implications

This study highlights the potential of targeting Tregs and TG2 inhibitors as promising therapeutic strategies for celiac disease. By modulating immune tolerance and reducing intestinal damage, these interventions can improve patient outcomes, reduce symptoms, and enhance quality of life, offering alternatives or adjuncts to the current gluten-free diet standard of care.

Limitations and recommendations

The systematic review shows that combining TG2 inhibitors with Tregs provides potential therapeutic options beyond restrictive gluten diets for treating celiac disease pathologies. Medical investigations demonstrate that TG2 inhibitors help reduce intestinal inflammation and enhance Treg activities for sustained gluten antigen tolerance to enable better disease control. However, the findings have limitations because of the use of limited data from large clinical studies, together with variations in testing approaches and preclinical testing with animals and initial human trials that prevent results from reaching practical application. Adequately powered randomized controlled trials using uniform outcome measures are needed when examining the long-term effectiveness of immunomodulatory programs for clinical application.

## Conclusions

The study concluded that both Treg therapy and TG2 inhibitors demonstrate promising therapeutic potential in the management of celiac disease. These treatment approaches aim to modulate the dysregulated immune response characteristic of the condition, offering a targeted strategy to restore immune tolerance to gluten. Tregs play a pivotal role in suppressing inappropriate immune activation, thereby potentially reducing intestinal inflammation and mucosal damage. Similarly, TG2 inhibitors may prevent the modification of gluten peptides, which is a key step in triggering the autoimmune response in genetically predisposed individuals. The immunomodulatory effects observed through these therapies suggest the emergence of novel, mechanism-based treatment avenues that go beyond the traditional gluten-free diet, which remains the cornerstone of current management. However, while preclinical data and early-phase trials provide a foundation of encouraging results, further clinical research is necessary to validate their long-term efficacy, safety, and applicability in diverse patient populations.
